# Posttransplant lymphoproliferative disorders in kidney transplant recipients: a retrospective cohort analysis over two decades in Hong Kong

**DOI:** 10.18632/oncotarget.18890

**Published:** 2017-06-30

**Authors:** Chi Yuen Cheung, Maggie Kam Man Ma, Ka Foon Chau, Wai Leung Chak, Sydney Chi Wai Tang

**Affiliations:** ^1^ Renal Unit, Department of Medicine, Queen Elizabeth Hospital, Hong Kong SAR, China; ^2^ Division of Nephrology, Department of Medicine, The University of Hong Kong, Queen Mary Hospital, Hong Kong SAR, China

**Keywords:** lymphoma, posttransplant lymphoproliferative disorders, kidney transplant

## Abstract

**Objective:**

To characterize the posttransplant lymphoproliferative disorders (PTLD) including the Epstein-Barr virus (EBV) status, histological subgroups, site of occurrence and the clinical outcome in the Chinese kidney transplant recipients.

**Methods:**

A retrospective cohort study of 1, 227 adult kidney transplant recipients who were followed up in two transplant centers in Hong Kong over two decades.

**Results:**

23 (1.9%) patients developed PTLD. Median duration from transplant to PTLD was 104 (5-252) months. Six patients (26.1%) had early PTLD and 17 (73.9%) had late PTLD. Ten (43%) developed PTLD >10 years after transplant. All patients in early PTLD group were EBV-positive. In the late PTLD group, 60% were EBV-negative and 40% EBV-positive. More than 90% of cases were monomorphic PTLD with majority being diffuse large B cell lymphoma. Bone marrow was the most common extranodal site. The overall treatment response rate was 52.2 %. None of the patients developed rejection or relapse after PTLD. At a median follow-up of 9 (1-79) months after PTLD, 18 patients died. Patient survival was 48% at 1 year and 30% at 3 years and death-censored allograft survival was 82% at 1year and 73% at 3 years.

**Conclusion:**

Late PTLD is common. Careful adjustment of immunosuppression, close monitoring of patients, increased awareness and early detection of the disease are essential.

## INTRODUCTION

Posttransplant lymphoproliferative disorders (PTLD) represent a heterogeneous group of lymphoid and plasmacytic proliferative diseases, occurring as a result of immunosuppressive therapy in patients who have received solid organ or bone marrow transplantation [1]. The disease can occur in a wide range of locations, which may be limited to nodal disease or disseminated to involve a variety of extranodal sites including the bone marrow, central nervous system, gastrointestinal system and the transplanted allograft [2, 3]. Different studies have shown that PTLD is one of the most common cancers in kidney transplant recipients. Moreover, presence of PTLD can reduce the patient and allograft survival [2-8].

Epstein-Barr virus (EBV) is a significant risk factor for the development of PTLD. However, a substantial portion of PTLD cases is not associated with EBV. The EBV-negative PTLD cases tend to occur late when compared with those EBV-positive cases [9]. It is still not known whether EBV-negative PTLD may actually represent another disease entity which requires more aggressive treatment. Previous studies showed conflicting data regarding the prognostic significance of the EBV status in PTLD [2, 10-16]. In addition, the subjects in most of these studies were from a heterogeneous group involving both adult and pediatric patients and recipients of different types of solid organ. Furthermore, only very few studies of kidney transplant recipients with PTLD were recruited from the Asian population, especially the Chinese patients and these data are usually restricted to small case series [8, 17-19]. The difference in EBV seropositivity and HLA frequencies between the Western and Asian population might influence the incidence, pattern and treatment outcome of PTLD in different geographical areas [17, 18]. The aim of this study was to characterize the PTLD including the EBV status, histological subgroups, site of occurrence and the clinical outcome in the adult kidney transplant recipients in Hong Kong over two decades.

## RESULTS

Total 1, 227 kidney transplant recipients had follow-up within the study period and 23 (1.9%) of them developed PTLD. Among the PTLD patients, 13 were males and 10 were females. The mean age at transplant was 42.3 +/- 12.0 years. The median duration from transplant to onset of PTLD was 104 (5-252) months. Six patients (26.1% of all PTLD cases) were classified as early PTLD and the median duration from transplant to PTLD in these patients was 9 [5-11] months. On the other hand, 17 patients (73.9%) were classified as late PTLD and the median duration from transplant to PTLD was 154 (23-252) months. Ten patients (43%) developed PTLD more than 10 years after transplant. Total 4 patients received anti-thymocyte globulin before PTLD: 3 given as induction therapy and 1 as treatment of acute rejection. The baseline demographics and clinicopathologic features of patients with PTLD in our cohort were summarized in Tables 1 and 2.

**Table 1:** Summary of the baseline demographics and clinicopathologic features of patients with post-transplant lymphoproliferative disorders

Patients with PTLD	n=23
Male, n (%)	13 (56.5)
Mean age at transplant (years)	42.3 +/- 12.0
Mode of dialysis, n (%)	
Peritoneal dialysis	11 (47.8)
Hemodialysis	6 (26.1)
Preemptive treatment	6 (26.1)
ESKD cause, n (%)	
Glomerulonephritis	13 (56.5)
Hypertension	5 (21.7)
Diabetes mellitus	2 (8.7)
Others	3 (13.1)
Deceased / Living, n (%)	20 (86.9) / 3 (13.1)
Year of transplantation	
2000 or before	14 (60.9)
2001-2010	9 (39.1)
2011-2015	0 (0)
Induction therapy, n (%)	
Anti-thymocyte globulin	3 (13.0)
Basiliximab	5 (21.7)
None	15 (65.3)
Maintenance immunosuppression, n (%)	
Prednisolone	21 (91.3)
Azathioprine	9 (39.1)
Mycophenolatemofetil	7 (30.4)
Cyclosporine	17 (73.9)
Tacrolimus	6 (26.1)
Sirolimus	2 (8.7)
Everolimus	2 (8.7)
Rejection therapy before PTLD, n (%)	
Yes	10 (43.5)
No	13 (56.5)
Mean age at PTLD (years)	51.5 +/- 14.4
Median duration from transplant to PTLD (months)	104 (5-252)
Year of PTLD	
2000 or before	4 (17.4)
2001-2010	5 (21.7)
2011-2015	14 (60.9)
Early PTLD / Late PTLD, n (%)	6 (26.1) / 17 (73.9)
Stage I and II / Stage III and IV, n (%)	7 (30.4) / 16 (69.6)
Histological classification, n (%)	
Polymorphic PTLD	1 (4.3)
Diffuse large B cell lymphoma	15 (65.5)
Burkitt lymphoma	1 (4.3)
NK cell lymphoma	1 (4.3)
T cell lymphoma	1 (4.3)
Mantle cell lymphoma	1 (4.3)
Plasma cell myeloma	2 (8.7)
Marginal zone lymphoma	1 (4.3)
Epstein-Barr virus status of the tissue, n (%)	
Positive	12 (52.1)
Negative	9 (39.2)
Not determined	2 (8.7)
Location of PTLD, n (%)	
Lymph node	8 (34.8)
Bone marrow	7 (30.4)
Kidney graft	2 (8.7)
Brain	4 (17.4)
Gastrointestinal tract	4 (17.4)
Tonsil	2 (8.7)
Liver	2 (8.7)
Spleen	2 (8.7)
Nasopharynx	3 (13.0)
Others	3 (13.0)

Values expressed as mean ±SD, median (range) or number (percentage).ESKD: end stage kidney disease; PTLD: post-transplant lymphoproliferative disorders.

**Table 2:** Clinicopathologic features and treatment outcome of individual patient with post-transplant lymphoproliferative disorders

Patent	Gender/age (Tx)	Primary disease	Histology	EBV	Sites	Time (month)	IS	Reject	Treatment (remission)	Outcome (month)
1	F/29	GN	DLBCL	+	graft	5	Pred+Aza+CsA ATG	+	Surgery (CR)	Died (49) IHD
2	M/24	HT	DLBCL	+	brain	9	Pred+CsA	+	RT + CHOP (NR)	Died (6) cancer
3	M/57	DM	Polymorphic	+	graft	9	Pred+MMF+CsA	+	R (PR)	Died (8) sepsis
4	M/25	GN	DLBCL	+	LN, NP	9	Pred+Tac+Siro Basiliximab	+	R-CHOP (NR)	Died (3) sepsis
5	F/49	GN	DLBCL	+	brain	10	Pred+MMF+FK ATG	+	RT (NR)	Died (4) cancer
6	M/43	GN	NK cell	+	BM, nasal septum	11	Pred+Aza+CsA	+	CHOP (NR)	Died (2) cancer
7	M/36	HT	Mantle cell	-	LN, BM, liver, spleen	23	Pred+MMF+Tac	-	R-CHOP (PR)	Died (13) sepsis
8	M/42	GN	DLBCL	-	liver	45	Pred+Aza+CsA ATG	-	R-CHOP (CR)	Alive (79)
9	M/38	GN	T cell	-	BM, NP, colon	54	Pred+MMF+Tac Basiliximab	+	CHOP (NR)	Died (14) sepsis
10	F/54	HT	myeloma	ND	BM	68	Pred+Aza+CsA Basiliximab	-	Chemotherapy (CR)	Died (14) IHD
11	F/45	unknown	DLBCL	-	LN, small bowel	87	Pred+MMF+Tac	-	Surgery + R-CHOP (PR)	Died (4) sepsis
12	F/69	DM	DLBCL	-	LN, NP, BM	104	Pred+CsA	-	CHOP (NR)	Died (3) cancer
13	F/45	GN	DLBCL	+	LN, tonsils, BM, soft tissue mass	109	Pred+Aza+CsA	-	R-CHOP (CR)	Alive (29)
14	M/28	GN	DLBCL	-	BM, spleen	139	Pred+CsA	+	Surgery + R-CHOP (NR)	Died (1) sepsis
15	F/49	GN	DLBCL	-	LN, tonsils	154	Pred+Aza+CsA	-	R-CHOP (CR)	Alive (50)
16	F/40	GN	DLBCL	+	small bowel, colon	163	Pred+Tac+Ever Basiliximab	+	Surgery + R (CR)	Died (40) sepsis
17	M/66	HT	myeloma	ND	BM	174	Pred+CsA+Siro Basiliximab	-	Thalidomide (NR)	Died (15) cancer
18	M/50	GN	DLBCL	-	LN, BM	192	Pred+CsA	-	R-chlorambucil (NR)	Died (1) sepsis
19	F/40	GN	DLBCL	+	brain	214	Pred+MMF+CsA	-	Conservative (NR)	Died (1) cancer
20	F/38	SLE	DLBCL	+	small bowel	215	Pred+CsA+Ever	-	Surgery + R-CHOP (CR)	Alive (54)
21	M/28	unknown	DLBCL	+	brain	236	Pred+MMF+CsA	-	RT (NR)	Died (1) sepsis
22	M/35	GN	Marginal zone	+	LN	250	Pred+Aza+CsA	+	Reduce IS (CR)	Alive (65)
23	M/44	HT	Burkitt	-	soft tissue mass	252	Aza+CsA	-	RT + R-CHOP (CR)	Died (9) sepsis

PTLD: post-transplant lymphoproliferative disorders; Age (Tx): age at transplant; EBV: Epstein-Barr virus status of tissue; Time (month): time from transplant to onset of PTLD (months); IS: immunosuppression; Reject: history of rejection before PTLD; Outcome (month): outcome of treatment including cause of death and time from onset of PTLD to time of death or 31/12/2016.

M: male; F: female; GN: glomerulonephritis; HT: hypertension; SLE: systemic lupus erythematosus; DLBCL: diffuse large B cell lymphoma; Polymorphic: polymorphic PTLD; NK cell: NK cell lymphoma; Mantle cell: mantle cell lymphoma; T cell: peripheral T cell lymphoma; myeloma: plasma cell myeloma; ND: not determined; LN: lymph node; NP: nasopharynx; BM: bone marrow; Pred: prednisolone; Aza: azathioprine; MMF: mycophenolate mofetil; CsA: cyclosporine A; Tac: tacrolimus; siro: sirolimus; ever: everolimus; ATG: anti-thymocyte globulin; RT: radiotherapy; R: rituximab; CHOP: cyclophosphamide, doxorubicin, vincristine, prednisolone; CR: complete remission; NR: no remission; PR: partial remission; IHD: ischemic heart disease.

EBV status of PTLD histology was available in 21 patients (2 patients with plasma cell myeloma had unknown EBV status). Twelve cases (57.1%) were positive for EBV; 9 being DLBCL, 1 being polymorphic PTLD, 1 being NK cell lymphoma and 1 being marginal zone lymphoma. On the other hand, 9 cases were negative for EBV (6 DLBCL, 1 mantle cell lymphoma, 1 peripheral T cell lymphoma and 1 Burkitt lymphoma). All 6 patients in early PTLD group were EBV-positive. In the late PTLD group, 60% (9/15) were EBV-negative and 40% (6/15) were EBV-positive. The baseline demographics and disease characteristics, categorized by EBV status, was shown in Table 3.

**Table 3:** Patient demographics and clinical characteristics stratified by EBV status

	EBV-positive (n=12)	EBV-negative (n=9)	P value
Male, n (%)	6 (50.0)	6 (66.7)	0.66
Deceased / living, n (%)	11 (91.7)	7 (77.8)	0.55
Mean age at transplant (years)	37.8 +/- 10.1	44.6 +/- 11.4	0.16
Mean age at PTLD (years)	46.3 +/- 12.4	54.3 +/- 14.0	0.18
Median duration from transplant to PTLD (months)	60 (5-250)	104 (23-252)	0.48
Extranodal disease, n (%)	11 (91.7)	9 (100)	1.00
Central nervous system, n (%)	4 (33.3)	0 (0)	0.10
Bone marrow involvement, n (%)	2 (16.7)	5 (55.6)	0.16
Graft involvement, n (%)	2 (16.7)	0 (0)	0.49
Ann Arbor stage, n (%)			0.64
I/II	5 (41.7)	2 (22.2)	
III/IV	7 (58.3)	7 (77.8)	
PTLD subtype, n (%)			0.99
Polymorphic PTLD	1 (8.3)	0 (0)	
DLBCL	9 (75.0)	6 (66.7)	
Burkitt lymphoma	0 (0)	1 (11.1)	
Mantle cell lymphoma	0 (0)	1 (11.1)	
NK cell lymphoma	1 (8.3)	0 (0)	
Peripheral T cell lymphoma	0 (0)	1 (11.1)	
Marginal zone lymphoma	1 (8.3)	0 (0)	
Immunosuppression at diagnosis, n (%)			
Prednisolone	12 (100)	7 (77.8)	0.17
Azathioprine	4 (33.3)	4 (44.4)	0.67
Mycophenolate mofetil	4 (33.3)	3 (33.3)	1.00
Cyclosporine A	9 (75.0)	6 (66.7)	1.00
Tacrolimus	3 (25.0)	3 (33.3)	1.00
Sirolimus	1 (8.3)	0 (0)	1.00
Everolimus	2(16.7)	0 (0)	0.49
Use of Rituximab in PTLD treatment, n (%)	5 (41.7)	6 (66.7)	0.38

Values expressed as mean ±SD, median (range) or number (percentage).

DLBCL: diffuse large B cell lymphoma; PTLD: post-transplant lymphoproliferative disorders.

Most cases (91.3%) in our series were monomorphic PTLD with majority being diffuse large B cell lymphoma (DLBCL, n=15) (Table 1). On the other hand, none of the kidney transplant recipients developed classical Hodgkin lymphoma type PTLD. Lymph nodes were the most common location of PTLD (34.8%), followed by bone marrow (30.4%), brain and gastrointestinal tract (17.4%). All patients except the one with nodal marginal zone lymphoma had extranodal involvement of PTLD. More patients (16/23, 69.6%) presented with late-stage (III or IV) PTLD than early-stage disease (7/23, 30.4%) in our cohort.

Reduction in immunosuppression was included as part of the first line therapy in all patients with PTLD. Among these patients, 11 patients had mTOR inhibitor conversion (7 converted to sirolimus and 4 to everolimus) together with CNI minimization or elimination. None of the patients suffered from any serious side effects of mTOR inhibitors. Two patients were treated with reduction in immunosuppression alone; including one patient (Patient 22) who was diagnosed with nodal marginal zone lymphoma (EBV-positive) and had complete regression of lesion, and one patient (Patient 19) who was too ill to receive other forms of treatment. Sixteen patients received chemotherapy. Rituximab treatment was administered, either alone or in combination with chemotherapy, in 12 patients. Four patients with brain or soft tissue involvement received radiotherapy and 5 patients required surgery (1 with graft PTLD received graft nephrectomy, 1 with spleen involvement had splenectomy and 3 with bowel involvement had laparotomy and bowel resection).

The overall treatment response rate (including complete and partial remission) was 52.2 (12/23) %. None of the patients had PTLD relapse within the study period. At a median follow-up of 9 (1-79) months after PTLD, 18 patients died (6 due to PTLD progression, 10 due to sepsis and 2 due to ischemic heart disease). The median duration from diagnosis of PTLD to death was 5 months (1 month to 49 months). Overall patient survival after diagnosis of PTLD was 48% at 1 year and 30% at 3 years. For those who died from cancer progression, 4 had DLBCL (3 of them with cerebral involvement), 1 had NK cell lymphoma, and 1 had plasma cell myeloma. Among the 15 patients who received mTOR inhibitors after PTLD (11 from conversion while 4 already on mTOR inhibitors before cancer), 11 patients died (9 due to sepsis, 1 due to ischemic heart disease, 1 due to cancer progression). On the other hand, 7 of the 8 patients who were not on mTOR inhibitors died (5 due to cancer progression, 1 due to sepsis and 1 due to ischemic heart disease). Five patients died of sepsis during the treatment of PTLD: 2 had Pseudomonas aeruginosa septicemia, 1 had extended-spectrum beta-lactamase-producing Escherichia coli septicemia, 1 had candidemia and 1 had Pneumocystis carinii pneumonia.

If we classified the patients according to the EBV status, there was no significant difference in patient survival between those with EBV-positive PTLD and EBV-negative PTLD (P=0.95). If we examined our cohort again according to the onset of disease, all 6 patients with early PTLD died. On the other hand, 5 patients with late PTLD survived at the end of the study (4 with DLBCL and 1 with marginal zone lymphoma). The median follow-up duration in these 5 survivors was 54 (29-79) months.

Four patients lost their grafts during the study period (1 had graft nephrectomy and the other 3 already had chronic allograft nephropathy with poor renal function before PTLD). None of the patients developed acute rejection after PTLD diagnosis and titration of immunosuppression. The death-censored allograft survival was 82% at 1year and 73% at 3 years.

## DISCUSSION

Our previous registry study has shown that non Hodgkin lymphoma (NHL) is the most common post-transplant cancers in the Chinese kidney transplant recipients in Hong Kong. The incidence rate of NHL is 1.2 cases per 1000 patient-years. Compared with the general population, we found approximately 15 times increased risk of NHL after kidney transplantation. Moreover, the mortality risk of NHL is also markedly elevated (standardized mortality ratio 18.2 with 95% confidence interval 14.0-23.8) [4]. Since PTLD is such an important comorbidity after kidney transplant, we performed this retrospective cohort study in order to characterize the PTLD into subgroups according to the EBV status, histology, site of occurrence and the clinical outcome in our kidney transplant recipients. In fact our study reports the largest cohort of Asian patients with PTLD to date.

Previous study reported that PTLD occurred at a median of 6 months after solid organ transplantation [20], although recent data show that this interval has increased to 48-81 months [11, 21]. In our cohort, the median time from transplantation to PTLD diagnosis was even longer (104 months). As a result, a higher portion of our PTLD cohort belonged to the late PTLD group when compared with other study (73.9% vs 60%) [11]. In addition, 43% of the PTLD patients in our cohort, as opposed to only 15-30% in other series, were diagnosed to have PTLD more than 10 years after transplant [11, 21]. Late PTLD is becoming more common in kidney transplant recipients because of the improved patient and graft survival in recent years. Early PTLD was associated with pre-transplant EBV seronegativity and use of T cell-depleting antibodies. On the other hand, late PTLD was associated with the prolonged use of potent immunosuppressive agents such as CNI [22]. Recognition of late PTLD is important because it has been shown to be associated with poor prognosis in different studies [2, 12, 23].

Although there is a strong association between EBV and PTLD, different studies showed that up to 33-48% of PTLD were EBV-negative [9, 10]. In our cohort, approximately 43% of PTLD cases were EBV-negative. However, none of these EBV-negative cases occurred within the first year of transplant. Similar to other series [9-11, 21], EBV-negative PTLD seems to present late after transplant when compared with the EBV-positive cases (104 vs 60 months). Some studies reported that EBV-negativity was associated with poor response to rituximab treatment and inferior patient survival [12, 16]. However similar association was not shown in other retrospective and prospective studies [10, 11, 13-15]. Luskin et al. found that the EBV status had no significant impact on patient survival and response to initial PTLD therapy [10]. In our study, we also could not show any significant difference in the overall survival of PTLD patients with EBV-negative versus EBV-positive disease.

Minimization of immunosuppression is the mainstay of treatment after PTLD diagnosis. However, there is no consensus on how the immunosuppressive drugs should be adjusted. One commonly used strategy is to eliminate the CNI completely. However, Serre et al. showed that maintaining CNI at a lower dose after PTLD could reduce the risk of developing de novo anti-HLA antibodies and humoral rejection while absence of CNI in the maintenance immunosuppressive regimen was an independent risk factors for allograft loss. In addition, CNI maintenance was neither associated with worse progression-free survival nor higher mortality [24]. mTOR inhibitor has both immunosuppressive and antitumor effects. It was able to suppress growth of cells derived from PTLD *in vitro* at a dose that could prevent allograft rejection [25]. Total 15 patients were on mTOR inhibitors after PTLD (4 on mTOR inhibitors before PTLD and 11 converted from azathioprine/mycophenolate) together with CNI minimization or elimination. None of the patients in our cohort developed acute or chronic rejection after PTLD. With the low number of patients on mTOR inhibitors and limited follow-up duration, it remains to be seen whether conversion to mTOR inhibitors after PTLD development can have any benefit in the long-term patient and graft survival.

The treatment response rate and the patient survival varied widely among different studies. In our cohort, the overall treatment response rate was around 52%, which was comparable with other large studies [10, 21, 23]. However, the mortality was high when compared with other series [2, 11, 21]. Many patients died of infection shortly after chemotherapy. It may be worthwhile to have a greater reduction of immunosuppression during the treatment phase even though there is a higher risk of acute rejection. Moreover, there were different poor prognostic factors in our cohort. Extranodal disease was present in almost all patients. Bone marrow involvement was the most common extranodal site of PTLD in our study (30.4%), which was followed by brain and gastrointestinal tract involvement (17.4%). Different studies have reported that central nervous system and bone marrow involvement are 2 important poor prognostic factors of PTLD [2, 10, 11]. Moreover, more than 90% of PTLD cases in our series were monomorphic disease which was also another well-known prognostic indicator [12, 19, 26]. Finally, approximately 70% of the patients in our study presented with late-stage disease (Stage III and IV).

This study had several strengths. Firstly, all cases of PTLD were captured from the Hong Kong Renal Registry which was an accurate and up-to-date record of all comorbidities and complications of patients receiving renal replacement therapy in Hong Kong under HA. The diagnosis of PTLD was subsequently verified with the histology and other relevant information from the patients’ medical record. Secondly, we covered all kidney transplant recipients in 2 large transplant centers in Hong Kong over 2 decades, resulting in over 1200 adult kidney transplants included. Moreover, prolonged follow-up duration also enabled us to include those patients with very late PTLD (onset >10 years after transplant). Finally, EBER *in situ* hybridization results were available in nearly all biopsy samples in our cohort. Despite of these, our study also had some limitations. The retrospective nature of the study, the small number of patients diagnosed to have PTLD and the lack of uniform treatment assignment over 20 years have limited our ability to determine the significant risk factors and the prognostic indicators of various subgroups of PTLD in our population.

In conclusion, late PTLD is common and has become an important comorbidity in kidney transplant recipients. Careful adjustment of immunosuppression, close monitoring of patients, increased awareness and early detection of the disease are required. Prospective studies involving more patients, longer follow-up duration and standardized treatment protocol are necessary for further understanding the different subtypes of PTLD including the appropriate treatment in our population.

## MATERIALS AND METHODS

This was a retrospective cohort study of all adult kidney transplant recipients (>=18 years old) who had been followed up in the two large transplant centers in Hong Kong, namely Queen Elizabeth Hospital and Queen Mary Hospital, between the period 1^st^January 1994 and 30^th^ June 2015 and were diagnosed to have PTLD. The diagnosis of PTLD was confirmed by histological assessment on incisional/excisional biopsies or resection of tissues. Bone marrow involvement was determined by bone marrow aspiration and trephine biopsy. The cases were then reviewed and classified according to the 2008 WHO classification: (1) early lesions (plasmacytic hyperplasia and infectious mononucleosis-like PTLD); (2) polymorphic PTLD; (3) monomorphic PTLD (B cell and T cell/NK cell types) and (4) classical Hodgkin lymphoma type PTLD (1). Since we would like to study all lymphomas and early PTLD in the kidney transplant recipients, indolent lymphomas were also included in our study although they are not defined as PTLD according to WHO classification. In addition, *in-situ* hybridization for EBV-encoded RNAs (EBER) was performed to confirm diagnosis of EBV-associated disease. Clinical stage was defined according to Ann Arbor criteria. Early PTLD was defined as PTLD diagnosed within the first year of transplantation while all other cases were defined as late PTLD.

All patients with PTLD were identified from the Hong Kong Renal Registry database. The Renal Registry is a direct, online, computerized registry system developed by the Central Renal Committee, Hospital Authority (HA), to collect data of all patients who received renal replacement therapy under HA. The data is entered directly by renal doctors or nurses of the individual renal unit and each unit can access their own patient data [27]. All basic demographic and clinical data were extracted from patients’ medical record. This study was approved by the research ethics committee.

All patients received calcineurin inhibitor (CNI), either tacrolimus or cyclosporine, as part of the maintenance immuosuppressive regimen after kidney transplant. When cancer was diagnosed, the dose of immunosuppressive drugs would be reduced. Since 2006, those patients with cancers would have their immunosuppressive regimen converted to inhibitor of mammalian target of rapamycin (mTOR inhibitor, either sirolimus or everolimus) – based therapy which consisted of abrupt withdrawal of either mycophenolate or azathioprine together with CNI minimization or elimination. However, mTOR inhibitor would not be prescribed for those who refused conversion therapy. When PTLD was diagnosed, the target cyclosporine trough level was 50-80 ng/mL and the target tacrolimus trough level was 3-5 ng/mL. For mTOR inhibitor titration, the target sirolimus trough level was maintained at 5-10 ng/mL while the target everolimus trough level was 3-8 ng/mL.

SPSS (SPSS 20.0, Inc., Chicago, IL USA) was used to perform the statistical analyses. Categorical data was expressed as percentages while continuous data was expressed as mean +/- standard deviation (SD) or median (range). Categorical data were compared with chi-square or Fisher’s exact tests while continuous data were compared with t-test or Mann-Whitney U test. Patient survival was calculated from the time of PTLD diagnosis to the time of death or 31^st^ December 2016. Graft survival was calculated from the time of PTLD to the time of resumption of dialysis or patient death or 31^st^December 2016. Kaplan-Meier method was used to plot survival and log-rank test was used to compare the survival between groups. P value <0.05 was considered statistically significant in this study.
